# Acceptability, Usability, and Views on Deployment of Peek, a Mobile Phone mHealth Intervention for Eye Care in Kenya: Qualitative Study

**DOI:** 10.2196/mhealth.4746

**Published:** 2016-05-09

**Authors:** Vaishali Lodhia, Sarah Karanja, Shelley Lees, Andrew Bastawrous

**Affiliations:** ^1^ London School of Hygiene and Tropical Medicine London United Kingdom; ^2^ AMREF Nairobi Kenya; ^3^ London School of Hygiene and Tropical Medicine Department of Global Health and Development London United Kingdom; ^4^ London School of Hygiene and Tropical Medicine Clinical Research Department, International Centre for Eye Health London United Kingdom

**Keywords:** mobile phone, mHealth, qualitative, ophthalmic testing, acceptability, usability, Kenya

## Abstract

**Background:**

The Portable Eye Examination Kit (Peek) is a mobile phone–based ophthalmic testing system that has been developed to perform comprehensive eye examinations. Shortages in ophthalmic personnel, the high cost, and the difficulty in transporting equipment have made it challenging to offer services, particularly in rural areas. Peek offers a solution for overcoming barriers of limited access to traditional ophthalmic testing methods and has been pilot tested on adults in Nakuru, Kenya, and compared with traditional eye examination tools.

**Objective:**

This qualitative study evaluated the acceptability and usability of Peek in addition to perceptions regarding its adoption and nationwide deployment.

**Methods:**

Semistructured interviews were conducted with patients and analyzed using a framework approach. This included analysis of interviews from 20 patients, 8 health care providers (HCPs), and 4 key decision makers in ophthalmic health care provision in Kenya. The participants were purposefully sampled. The coding structure involved predefined themes for assessing the following: (1) the context, that is, environment, user, task, and technology; (2) patient acceptability, that is, patients' perceived benefits, patient preference, and patient satisfaction; (3) usability, that is, efficiency, effectiveness, learnability, and flexibility and operability of Peek; and (4) the benefits of Peek in strengthening eye care provision, that is, capabilities enhancer, opportunity creator, social enabler, and knowledge generator. Emerging themes relating to the objectives were explored from the data using thematic analysis.

**Results:**

Patients found Peek to be acceptable because of its benefits in overcoming the barriers to accessing ophthalmic services. Most thought it to be fast, convenient, and able to reach a large population. All patients expressed being satisfied with Peek. The HCPs perceived it to satisfy the criteria for usability and found Peek to be acceptable based on the technology acceptance model. Peek was also found to have features required for strengthening ophthalmic delivery by aiding detection and diagnosis, provision of decision support, improving communication between provider and patient and among providers, linking patients to services, monitoring, and assisting in education and training. Some of the deployment-related issues included the need for government and community involvement, communication and awareness creation, data protection, infrastructure development including capacity creation, and training and maintenance support.

**Conclusions:**

According to all parties interviewed, Peek is an acceptable solution, as it provides a beneficial service, supports patients' needs, and fulfills HCPs' roles, overall contributing to strengthening eye health.

## Introduction

### Background

The estimated number of visually impaired people worldwide is 285 million, of which 39 million are blind [1]. Up to 80% of global visual impairment is preventable [1].The burden is unequally distributed, with the largest proportion living in low-income nations of Africa and Asia [2]. Loss of sight is associated with considerable emotional, social, and economic consequences, especially among the poor [3,4]. One of the biggest challenges to reducing the burden of visual impairment is the significant shortage of ophthalmic health care [5]. Ophthalmic testing equipment is often expensive, bulky, and immobile, making it difficult to deploy an ophthalmic service in rural areas, particularly where there are fewer ophthalmic professionals.

Tackling avoidable vision loss requires strengthening of health systems (HSs) in order to achieve universal access to ophthalmic services. This has been a major focus of the global ophthalmic public health community and a key goal of the recent World Health Organization (WHO) global action to improve eye health for everyone over the next 5 years, building on the principles of VISION 2020 [6].

Mobile health (mHealth) refers to the use of mobile technology, such as mobile phones (MPs), to provide health services. Mobile health is a growing field and its potential in improving health and health care delivery has been well demonstrated [7-9]. There are significant opportunities to leverage the benefits of mHealth in expanding health care delivery with the increasing uptake of MPs in the developing world [10]. Furthermore, the scope of mHealth has increased in recent years with the introduction of smartphones, which offer enhanced functionality and user interfaces over traditional multimedia devices.

In Africa, smartphones are becoming more affordable, driven by greater competition among operators and manufacturers. Smartphone subscriptions in Africa have been forecast to increase from 79 million to 412 million between 2012 and 2018 [11]. Nevertheless, the evidence base for smartphone use in health care is lacking, particularly contextual, process and health outcome evaluations in low- and middle-income countries [12-16].

**Figure 1:**
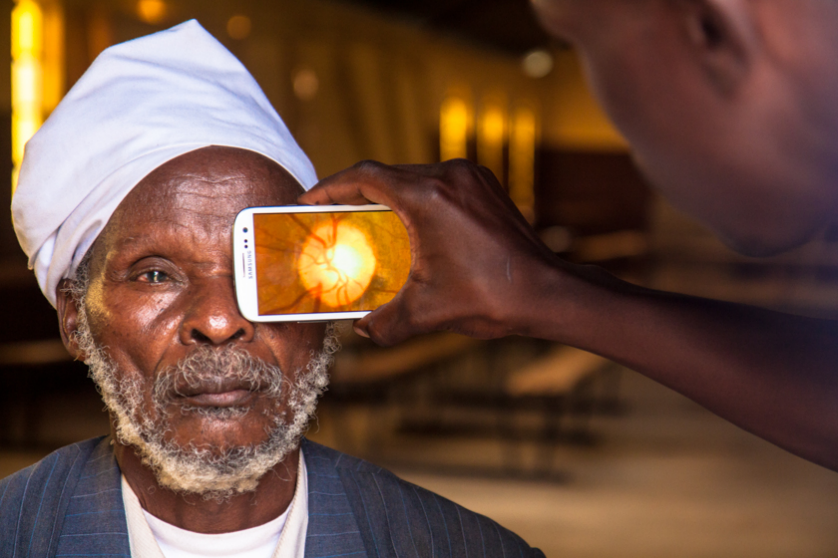
Retinal imaging by Peek.

**Figure 2:**
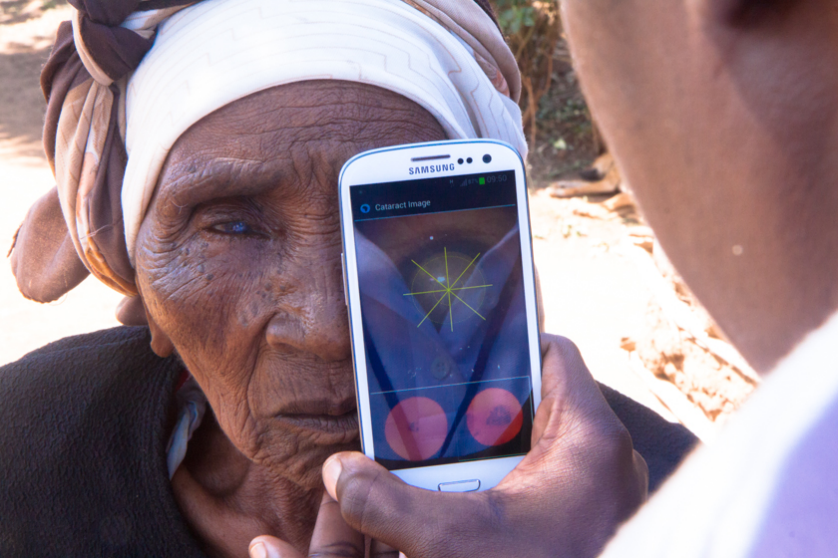
Peek for cataract testing outside patients home.

This study aimed to evaluate a smartphone-based ophthalmic examination system with clip-on hardware, the Portable Eye Examination Kit (Peek), which has been developed and introduced as a user-friendly and affordable alternative to perform comprehensive ophthalmic examinations. Figures 1 and 2 show how Peek is being used to take a fundal image and to examine a cataract. Peek offers a potential solution to overcoming barriers of traditional ophthalmic testing methods and thereby contributes to the VISION 2020 goals [17].

### Objectives

This qualitative study was carried out to assess patients', health care providers' (HCPs'), and stakeholders' (decision makers in ophthalmic service provision are referred to as “stakeholders”) perspectives on the adoption of Peek for improving the provision of ophthalmic services in Kenya. This included a formative evaluation of the acceptability and usability of Peek compared with traditional methods of ophthalmic testing. In addition, its potential for strengthening ophthalmic services and potential barriers and facilitators to adoption and deployment of the technology were explored.

## Methods

This study was undertaken within the follow-up phase of the Nakuru Eye Disease Cohort Study in central Kenya, a population-based study, which recruited 5000 individuals from 100 clusters [18].

The sampling strategy for this qualitative study involved purposeful sampling, and the patient sample was chosen from the 100 clusters based on varying sex, age, geographical location, educational levels, and income. The purpose was to maximize diversity and capture common themes relating to the intervention across a range of participants with differing characteristics [19]. Nakuru district was chosen because it offers a diverse population in terms of ethnicity and economic activities [18].

The qualitative study consisted of semistructured interviews with all HCPs (ie, 8) recruited for testing Peek, patients (ie, 40) examined with Peek, and key stakeholders (ie, 4) involved in shaping ophthalmic provision in Kenya and were chosen from the ministry of health, an ophthalmic teaching hospital, and selected nongovernmental organizations (NGOs). All patients underwent visual acuity (VA) testing using Peek at their homes, whereas fundal images were taken at a temporary clinic in that cluster. At this clinic, they also underwent repeat VA and funduscopic examinations using traditional equipment.

Four interviewers were trained to conduct the interviews and were provided semistructured interview guides. All interviews were audio recorded with the consent of all participants. The patient interviews were conducted in Kiswahili and then transcribed and translated. Conversely, the HCP and stakeholder interviews were conducted in English.

The Nakuru Eye Disease Cohort Study was approved by the African Medical and Research Foundation ethics board, which included pilot testing of Peek and conducting interviews as part of the larger project. Full written consent was obtained from all parties including the patients, HCPs, and stakeholders before each interview and was available in Kiswahili and English.

The interview transcripts were analyzed using NVivo version 10 [20]. A framework analysis approach was used, creating predefined themes for the coding structure. The coding framework was guided by theoretical constructs from literature on mobile usability, acceptability of technology, and previous mHealth reviews and literature on assessing benefits of mHealth in strengthening health care delivery [21-34].

To assess the acceptability and usability of Peek, it was deemed important to first understand the context within which it is to be implemented [21-23]. The analysis of the context was therefore carried out through a coding framework proposed by a qualitative review of mobile usability studies, which took into account the environment, user, task, and technology [21]. The themes involved assessing patients' and HCPs' perceptions of (1) ophthalmic service provision; (2) the barriers to seeking and accessing ophthalmic services, that is, environment; (3) HCP role and experience, that is, user; (4) understanding the purpose of Peek, that is, task; and (5) familiarity and views regarding mobile technology.

The coding for assessing patient acceptability of the Peek testing process and its functionality was informed by the definition of acceptability by Ayala and Elder (2011) [24]. They proposed that acceptability refers to determining how well an intervention will be received by the target population and the extent to which the new intervention or its components meet the needs of the target population and organizational setting. The themes included assessing (1) patients' perceived benefits of Peek, (2) patient satisfaction, and (3) patient preference.

The International Organization for Standardization defines usability as the extent to which a product can be used by specific users to achieve specific goals with efficiency, effectiveness, and satisfaction in a specified context of use [25]. A number of usability dimensions have been proposed by Coursaris and Kim (2006) in their qualitative review of mobile usability studies, which informed the coding framework for assessing usability of Peek by HCPs [21]. The themes included (1) efficiency, (2) effectiveness, (3) learnability, and (4) flexibility and operability.

The Technology Acceptance Model (TAM) and its extension, TAM2, were used as a guide to assess user acceptability of the technology [26-29]. This proposes that acceptability or prediction of use of a technology depends on the attitude toward it, which is a function of ease of use and perceived usefulness. The analysis of perceived usefulness was informed by assessing HCPs' perceptions of the benefits. Several studies to date have looked at the benefits of mHealth, and several frameworks have been proposed for assessing benefits in strengthening health care provision [30]. This analysis has therefore adapted a model based on a combination of four frameworks to appraise the potential benefits of Peek in strengthening eye care delivery. The four main themes of the framework were adapted from the Information Communication Technology for Healthcare Development Model [31]. These are capabilities enhancer, social enabler, opportunity producer, and knowledge generator. The subthemes were based on three other frameworks proposed for guiding assessment of mHealth in strengthening HSs [9,30,32,33]. The components chosen for the framework are also in line with the categories of mHealth initiatives established by the WHO [34].

A thematic analysis was also conducted to explore emerging themes and subthemes related to the study objectives that were inferred from the data [35-37]. Analysis was conducted until saturation was reached, which was 20 patient interviews, all 8 HCP interviews, and all 4 stakeholder interviews. After this, the coding was summarized and modified, and connections were made between related themes and between the 3 groups of interview participants.

## Results

A summary of all the themes discussed in this section is given in Textbox 1.

**Results section summary.**

Contextual factors

- Environment
  - Patient demographics
  - Barriers to seeking and accessing services
    - Cost
    - Lack of ophthalmic facilities, qualified providers, and support
    - Time
    - Lack of awareness
- User
  - Role and experience of health care providers
- Task
  - Patient's and health care provider's understanding of Portable Eye Examination Kit
- Technology
  - Attitudes toward mobile phones

Patient acceptability

- Perceived benefits of Portable Eye Examination Kit
- Patient preference
- Patient satisfaction

Usability dimensions

- Efficiency
  - Time
  - Multitasking
  - Portability and convenience
  - Cost
- Effectiveness
- Learnability
- Flexibility and operability

Benefits

- Capabilities enhancer
  - Detection and diagnosis
  - Provider performance
  - Decision support
- Social enabler
  - Provider-to-patient communication
  - Provider-to-provider communication
- Opportunity producer
  - Linkage of patients to ophthalmic provision
  - Monitoring and surveillance
- Knowledge generator
  - Training and education

### Contextual Factors

#### Patient Demographics

The number of males and females was equal and the ages of the patients ranged from 50 to 77 years. The educational levels varied from no education to primary, secondary, and tertiary education and were evenly represented in the sample of patients. With regard to occupation, 11 patients were farmers, 2 teachers, 2 businessmen or businesswomen, an engineer, an industrial chemist, a civil servant, and a secretary. Apart from one businessman who reported an annual income of 4,000,000 Kenyan shillings (KES), incomes varied mainly from 1000 to 50,000 KES per month with an average of 16,000 KES.

#### Environment (Patients' and HCPs' Perceptions of Current Eye Service Provision and Perceived Barriers to Seeking and Accessing Eye Services)

##### Cost

Six of the HCPs and almost all patients stated seeking ophthalmic services as unaffordable when referring to having to pay for hospital bills and transport.

...seeking eye treatment is quite expensive. Then again, I was not in a position to seek treatment. As farmers, we have low standards of living and therefore cannot afford to seek regular eye healthcare. We only go to hospitals when eye problems persist.

Patient #23, male

##### Lack of Ophthalmic Facilities, Qualified Personnel, and Support

The general opinion among the HCPs was that the availability of eye services in Kenya was inconsistent, with poorer provision in rural areas. The majority mentioned that patients had to travel long distances to access eye care, which was made more difficult because of poor infrastructure and roads. Another issue raised was scarcity of qualified ophthalmic personnel. Many described existing services as being overburdened as a result. With regard to prevention, most HCPs reported that they were not aware of any formal preventive measures put in place by the government in the region studied. The majority mentioned often taking the initiative to educate patients when seeing them.

Similarly, from patients' point of view, the government services in rural areas were reported to be limited to dispensaries with no specialist ophthalmic testing services, which only provide eye medications at a cost. Most patients also mentioned being on their own with little support posing a challenge for them to access treatment either because of the inability to access transport or due to having to prioritize other issues to sustain their livelihood. All those living in remote settings stated they were constrained from accessing more specialist services because of long distances.

In contrast, those living in Nakuru, an urban town, felt that ophthalmic facilities were generally accessible via government hospitals, private hospitals, opticians, and missionary hospitals.

Most patients are very far from health facilities, we have poor infrastructure and most clinics, health facilities that are near people don’t have eye clinic specialist, they just have a general doctor or clinician and that is all. So you find that most patients don’t get specific eye treatment.

HCP #6, female

It is far and then again, it is not easy to find. It is hard because even fare has to be considered and on top of that, there is the fee for treatment which is steep.

Patient #2, female

##### Time

Time was reported as a significant barrier to accessing eye services by 4 HCPs. Patients also had a similar opinion, especially those living in urban settings where long queues at government facilities were reported as a major obstacle because of difficulty taking time off work and potential loss of income.

Transportation and also long queues, time is also a factor because some are trying to work hard to see how the family could get along so they say the issue of the eye can be put aside, although he cannot see properly he says it’s an issue he can attend to later.

Patient #39, male

##### Lack of Awareness

Six HCPs mentioned lack of knowledge of eye conditions and lack of awareness of the importance of early detection and treatment as a barrier to patients seeking health care in both rural and urban settings. However, they perceived this to be more of a problem in rural areas due to lower educational levels and less exposure to health care in general.

Patients acknowledged that they had limited knowledge of eye problems. Most patients gave similar explanations for causes of eye problems and demonstrated a particularly limited understanding of chronic conditions. The most frequently mentioned causes, as perceived by patients, were poor hygiene, dust, smoke from cooking, direct sunlight, and unbalanced diet. A couple of participants mentioned “jerreri” as a cause, which means cataracts in their local language. Few patients mentioned other causes such as inherited diseases, work, alcohol, and smoking.

Interestingly, most patients revealed that they did not see the importance of regular eye checks despite being affected by changes in their vision. Only 4 patients brought up the importance of timely eye checkups as a means of preventing visual deterioration.

When asked about delays in seeking treatment, the most common reason given by patients and HCPs was that eye problems were not perceived to be serious enough to require urgent treatment, particularly when faced with barriers to accessing services. Furthermore, patients and HCPs perceived local myths and traditional practices in their communities to be a consequence of poor knowledge, leading to patients not seeking or accepting treatment. They highlighted the need for awareness creation through education and involvement of village chiefs.

...I thought that the problem was not very serious and I waited to see whether I would get better on my own but when that did not work, I sought medical treatment. This is because there are no mobile eye doctors like you doing rounds creating awareness on eye issues. Someone like I will wait until I am sick to seek treatment because there is no one giving people information to help prevent these problems.

Patient #9, male

They should receive help from people like chiefs who are more knowledgeable. They should be helped in accessing treatment. It can help in prevention. Because most people are now useless.

Patient #2, female

#### User (Role and Experience of Health Care Providers)

There were 6 male and 2 female HCPs consisting of ophthalmologists, ophthalmic clinical officers, and members of the advance team. The members of the “advance team” were responsible for tracking participants for the study enumeration, using Peek to test vision at patients' home and ophthalmic testing using traditional equipment in clinics. One of them had the additional responsibility of software maintenance. An ophthalmic nurse was also part of this team and was also involved in counseling and preparing patients for surgery. Most HCPs had no prior training in eye care before joining the Nakuru Eye Disease Cohort Study. Their experience in ophthalmic service provision was therefore mainly limited to the year during which the study took place, with the exception of the ophthalmologists, one of whom had 18 months and the other 4 years of experience.

#### Task (Understanding of Peek)

When asked to describe Peek and the examination process, the responses from HCPs varied based on their role and experience as ophthalmic providers. Most HCPs correctly mentioned that Peek incorporates several examinations in one device, thus enabling a basic eye examination comparable to traditional techniques. All the HCPs who were primarily responsible for providing outreach services described Peek as a tool for VA testing, and most needed prompting before mentioning its other uses in performing eye examinations such as anterior eye examination and fundoscopy. Most HCPs also highlighted Peek's capability for data analysis, information sharing, communication with colleagues, and other basic functionalities such as browsing, testing, and calling.

The patients interviewed demonstrated a good understanding of the technology and its purpose for ophthalmic testing and described it as an alternative and possible substitute for traditional eye examination. Two patients, however, were not aware that the MP was being used for eye examinations.

#### Technology (Attitudes Toward Mobile Phone Technology)

Patients, HCPs, and stakeholders all had positive attitudes toward MPs and smartphone technology. Mobile phones were referred to as innovative, advanced, new, and highly technological. They reported that the technology had made communication easier. Several patients revealed a familiarity in using MPs and felt that the attitude of the community toward MPs depends on exposure, awareness, and education. The HCPs and stakeholders had similar views and mentioned that MP use was widespread in the area. One HCP and patient reported that the use of MPs in Kenya was best known by the money transfer initiative called M-Pesa that has been adopted by a large proportion of the population. All participants were optimistic about the potential uses of MPs, especially smartphones, and portrayed enthusiasm for technology.

I think that the MP is a highly technological piece of equipment. It is very advanced.

Patient #27, female

In Kenya generally people are used to SMS, they are used to M-Pesa and the technology which is there is almost comparable to that. I think the kit generally most people are able to operate.

HCP #3, male

We all like new technology, we are all thirsty for new innovations in eye health because of the many challenges in service delivery.

Stakeholder #4

### Patient Acceptability

#### Benefits as Perceived by Patients

All patients perceived Peek to be beneficial as its portability brings examination and treatment closer to them. They perceived it as a way of overcoming many of the aforementioned barriers. The patients also suggested that it could increase detection of eye problems because it can reach a larger population. This could be achieved by providing earlier eye examinations for those who lack awareness or those unable to access existing eye services. The use of Peek was also seen to have the potential to increase awareness about eye conditions in general as it uses mobile technology, which is considered to be acceptable for patients. Many patients also deemed Peek to be efficient and economical for themselves and the HS because it saves time, costs less, and reduces the burden on health care personnel. When patients were asked about how long it took to receive an eye examination with Peek, 17 of the 20 patients recalled the time to be between 2 and 20 minutes. The other 3 patients did not mention an exact time. When they were asked about the duration of traditional eye examinations, the responses varied between 30 minutes and 4 hours.

#### Patient Satisfaction

All patients stated that they were satisfied with the service offered. Eighteen of 20 patients did not report concerns regarding the technology. Moreover, when asked about further comments about Peek at the end of the interview, the majority of patients stated that they were hoping for the service to be more accessible to them.

Nevertheless, some patients expressed potential doubts about their community's uptake of Peek. These will be discussed later in this paper.

#### Patient Preference

When asked about whether patients preferred traditional examinations or Peek, 10 patients expressed a preference for Peek, 7 stated no preference, and 3 preferred the traditional examination. The main reasons for preferring Peek were shorter examination time, simplicity, efficiency due to multiple examinations combined in one tool, being seen at home, and the increased potential coverage of the population in need. Those who did not express a preference stated that their decision would be dependent on the actual availability of the intervention. Two patients who preferred traditional examinations referred to the ease of reading larger letters. Another patient described clinic equipment as having fewer potential side effects, although he then conveyed his support for Peek to be incorporated into policy service provision in rural areas where the need was perceived to be greatest.

#### Patient Acceptability as Perceived by Health Care Providers

The HCPs also perceived Peek to be acceptable to patients. They reported that patients appreciate a service that is brought closer. Furthermore, according to HCPs patients were curious, interested, and willing to be examined by the new technology. Some HCPs also mentioned that the use of Peek helped overcome patients' fears related to being tested with traditional techniques, as mobiles are more familiar and therefore patients are likely to be more comfortable being tested with MPs.

The application being the first to debut in Kenya mostly using testing people with it, it is amazing and people are like they wish to be checked using the phone.

HCP #4, male

### Analysis of Health Care Providers' Usability of Peek

#### Usability Dimensions

Bearing in mind the context of use described earlier, an analysis of usability was carried out using the predefined usability dimensions, that is, efficiency, effectiveness, learnability, and flexibility and operability, as summarized in Table 1. While assessing efficiency, the following subthemes became apparent: speed, multitasking, convenience, and cost. Table 1 summarizes the analysis of perceptions of the HCPs regarding usability of Peek as per predefined usability dimensions.

**Table 1:** Usability dimensions.

Usability dimension		No. of HCPs^a^	Rationale given by HCPs	Benefits relating to usability dimension	HCP quotes
Efficiency					
	Time	8	Simple and easy to use, with less manual record keeping.	Ability to see more patients, early diagnosis, and treatment.	“ *...it will be more effective in that we will be able to get to see more patients with eye problems and in that case I will be able to solve them early enough and our patients will not have to go blind...”*
	Multitasking	6	Requires less equipment to navigate and manpower to conduct examinations.	Saves human resources.	“ *...you can multi task it by doing all the examination at the same place without moving just by the touch of the application, so it will make it better.”*
	Portability and convenience	7	Easier to carry around compared with traditional equipment.	Increase access and coverage in remote areas.	“ *...it’s portable and one can be able to access rural areas where infrastructure is poor so in terms of accessing those places you will be able to get people who could not think of getting help...”*
	Cost	6	Cheaper equipment (10,000-40,000 KES^a^compared with more than 1,000,000 KES for traditional equipment), transport, and negligible software costs and replacement costs.	Economic gains for patients and service provisions.	“ *...the cost of one Portable eye kit does like very many examination procedures compared to the machines so it makes it cheaper, two the cost of transport is cut down because I’ll be able to visit the client at his/her own convenience...”*
Effectiveness		6	Accurate, equal, or better than traditional equipment.	Ability to provide better analysis of findings, compared with the substitute.	“ *The phone is automatically accurate than the traditional type of equipment. PEEK is more advanced than the traditional equipment gives you the exact figures and images. It is very accurate. Excellent in fact”*
Learnability		8	Clear instructions; though useful, no expertise required.	Usable by less qualified HCPs with limited smartphone knowledge.	“ *...anybody as long as you have something in between your ears that is a brain then you can actually work. Because everything is just written and where it is not written you can actually see it everything is self-explanatory with algorithms.”*
Flexibility and operability		8	Quickly modifiable based on user feedback and robust technology.	User-friendly, easy to maintain, and meets different needs.	“ *...it is still open ended it is not closed so it is it is able to accommodate, new things and new ideas and new situations that may vary from one region to another from one country to another so it is adaptable.”*

^a^HCP: health care provider; KES: Kenyan shilling.

### HCPs' and Stakeholders' Perceptions of Benefits of Peek in Eye Care Delivery Using a HSs Approach

#### Capabilities Enhancer

##### Detection and Diagnosis

The HCPs and stakeholders believed that Peek can increase the chances of diagnosing eye problems and is thought to have the potential to be used as a screening tool to increase detection of poor vision. They perceived earlier detection to be beneficial in reducing the burden of blinding eye disease and thereby increasing general standards of living. Nevertheless, stakeholders stated that the success of Peek as a screening tool will depend on proven accuracy, sensitivity, availability, and ensuring high-quality service delivery.

Well the more sensitize a technology you have for detecting problems and the more easily available it is, it means you are going to start detecting many more patients and so that’s good for the patients so that more people get to know more earlier that they have a problem.

Stakeholder #1

##### Provider Performance and Decision Support

The opinion among HCPs and stakeholders was that Peek could allow for task shifting and improved human resource management by providing support to community health volunteers (CHVs). This was seen as a potential solution to fill in for the shortage of ophthalmic workforce. Peek was perceived to lead to improved outcomes of the services provided as a direct result from its user-friendly platform, inbuilt decision-support algorithms, and data analysis capabilities. These features of the application were also perceived to help in managing and organizing workload of HCPs, for example, by prioritizing referrals. Furthermore, the application was thought to have an impact on increasing HCP motivation and self-confidence in detecting and consequently managing eye problems.

I can even be able to collect, gather data from the field and it gives me some clear information on some decisions that I am about to make. The same way the smartphone and for example PEEK is doing; it is able to do some basic examination that is able to separate those who need to see a doctor urgently and those who do not need urgently.

Stakeholder #3

#### Social Enabler

##### Provider-to-Patient Communication or Client Education

Most stakeholders stated the value of Peek in providing instant feedback to patients through the images, which are immediately available on the phone. Stakeholders described its value in terms of explaining the diagnosis to the patient, reinforcing patient understanding, decision-making, and confidence. According to one stakeholder, this is further enhanced by the ability to contact relatives who are unable to make it to the clinic. Stakeholders highlighted that seeing an image of a damaged retina and optic nerve can help patients understand the seriousness of their problem and thus they will be more likely to urgently seek and comply with treatment as a result.

##### Provider-to-Provider Communication

Stakeholders referred to the potential role of Peek in enhancing communication between HCPs as a beneficial feature, for example, enabling remotely located ophthalmologists to provide less qualified HCPs with support in decision-making. Peek was also perceived to overcome current problems in data transfer by generating images in a format that can easily be transferred to other HCPs, which existing equipment does not allow. These qualities of Peek are further reported as vital for task shifting to be successful.

#### Opportunity Producer

##### Linkage of Patients to Ophthalmic Provision

Similar to HCP views, according to all the stakeholders interviewed, Peek was perceived as bringing service provision closer to patients who need it most. This was deemed possible by being able to conveniently and efficiently provide ophthalmic services in remote settings, thereby overcoming logistical issues in having to set up clinics. Additionally, 2 stakeholders commented on the ability of Peek to increase public confidence in ophthalmic workers and in service provision, which is currently challenged by poor uptake of eye services.

...those who are in the most remote areas who have the highest prevalence for blindness will now be linked to the health system and so people will be able to find them and treat them.

HCP #7, male

##### Monitoring and Surveillance

Features of Peek such as data storage and Global Positioning System tracking are thought to be desirable by stakeholders in strengthening monitoring and surveillance, thereby better contributing to policy-making and resource planning. Additionally, they perceive Peek to enhance follow-up by being able to locate patients easily.

#### Knowledge Generator

##### Training and Education

Peek was also deemed as a training opportunity by both HCPs and stakeholders, because discussing management of eye problems with qualified and experienced ophthalmic professionals is thought to increase knowledge and skills of those with limited training. Furthermore, according to stakeholders and HCPs, Peek offers an opportunity to educate and sensitize the population about eye health. Consequently, the overall opinion was that Peek contributes to increased patient awareness and knowledge.

### Analysis of Perceived Barriers, and Proposed Facilitators for Overcoming Potential Barriers to Adoption and Deployment

Neither the patients nor the HCPs reported any major obstacles with the use of Peek during the examination. However, the following themes emerged from all parties as potential system-related challenges in implementation that need to be considered for deployment.

#### Government Involvement

Lack of integration with the national health system and potential lack of government involvement were seen as major challenges to deploying Peek. Early involvement of government, policy makers, and health management teams in decision-making was therefore considered by stakeholders and HCPs to be essential to ensure sustainability of the program. These participants proposed working with the government at all stages from development to implementation. State involvement was also regarded as essential to gain public trust in the intervention; integrate services; and setting guidelines, standards, and protocols for national implementation and adoption.

#### Funding

Lack of funding was discussed as a barrier to deployment and sustainability of the program. Government support and partnership with NGOs was put forward as a solution to increase availability and affordability of Peek and integration with existing services. The new health restructuring in Kenya, where management has been devolved to county level, was perceived to be most likely beneficial in sustaining the intervention as resources are more likely to be spent where most needed. However, the priority given to eye health nationally was seen by the stakeholders to influence any future decisions about funding. Moreover, as indicated by one stakeholder, a cost-benefit analysis and evidence for effectiveness are essential for obtaining funding.

Other options suggested for funding were donor support for financing and other resources required for the program. Stakeholders also suggested that the government would financially benefit from adopting Peek, as it is perceived to be cost-effective compared with traditional ophthalmic testing methods.

From an economist point of view, I would say it is a good, it is a project worth financing.

Stakeholder #3

#### Communication and Technology Awareness

Although none of the patients expressed any reservations or fear of being examined by Peek, some mentioned that there is a possibility that certain people may not understand the purpose and value of the application. Another perceived barrier to adoption of using Peek was miscommunication. For instance, initially 1 patient reported having reservations about the use of Peek but was comfortable with it as soon as the examination steps were clearly explained. Furthermore, acceptance was also deemed to be governed by the level of education. Therefore, the importance of familiarity with MPs and the need for good communication on the utility of Peek were highlighted by several patients, HCPs, and stakeholders. Some examples were given for reasons of possible misunderstanding in the community, such as the phone being used to take patient's pictures instead of retinal images, cultural reservations about MPs, and fear of MPs having negative health effects.

Counselling and sharing with them and giving them reason as to why, especially if the patient needs examination, you just understand the patient and help the patient to understand.

HCP #6, female

#### Training and Product Support

A potential challenge mentioned by both stakeholders and HCPs is the need for setting up training for using Peek and product support if it were to be deployed sustainably. Consequently, they suggested the need to plan for a strong support team. Nevertheless, Peek was perceived as more sustainable than traditional equipment, with less likelihood of requiring replacement of expensive components. From patients' point of view, equipment quality was an important factor to ensure a high standard of care provision.

...it is more sustainable than the equipment we are providing and that is what I see. Because if these equipment breakdown, they have to be serviced and they have to buy spare parts, of which right now we have several equipment that are not working because of spare parts.

Stakeholder #4

#### Data Protection

According to HCPs and stakeholders, maintaining confidentiality of patient information is paramount and a potential barrier to sustainability and acceptability of the intervention. They proposed the need to ensure that a robust and secure data encryption system is in place. In addition, good communication was also reported as necessary to ensure that patients understand and are reassured about confidentiality. One stakeholder involved in building a central ophthalmic data collection unit, the Ophthalmic Service Unit designed to be linked to Hospital Management Information Systems, stated that it has been difficult to implement the system in Kenya. The suggestion for the implementation process was that it is important to link patient data, collected using Peek, to the HCPs' clinic as well as the central database for safekeeping.

Another issue raised was that mobile phone devices could be stolen when used in insecure remote areas, therefore reinforcing the need for robust security measures in addition to a data protection system.

#### Community Involvement

Stakeholders and HCPs described the benefits of training the local population for community mobilization. They suggested that training the local population to run the program will overcome any potential obstacles related to acceptability and sustainability. Patients saw the importance of community participation as key to building trust and confidence in the program and put the population at ease. From one stakeholder's point of view, getting public support is also very important to tackle cultural barriers. One patient referred to the M-Pesa service as an example of a program that has managed to drive community mobilization.

...early involvement and train locally available people to actually address some of those bugs that can arise that can cause a problem...

HCP #3, male

#### Increase in Demand for Ophthalmic Treatments

One obstacle mentioned was that the HS may not be able to cope with managing the increase in cases detected by Peek. A solution suggested by both HCPs and stakeholders to combat this problem is recruiting CHVs. The value of Peek in supporting HCPs who have limited training in eye care playing the role of CHVs has been highlighted throughout this study. Moreover, a stakeholder mentioned how Peek can be used to prioritize cases, which helps with shifting demand and coping with increasing workload. One stakeholder also proposed to introduce Peek to those trainees in the community who will become future HCPs.

#### Infrastructure

The accessibility of MPs and infrastructure supporting the use of mobiles was reported as being key for sustainability of Peek by HCPs and stakeholders. Infrastructure-related barriers raised were shortage of devices and poor mobile network provision and internet coverage, making it difficult to send across images and patient information to the central database as well as other HCPs for advice. They proposed partnering with and acquiring support from key network providers to increase availability and affordability of both the device and mobile data usage. Power shortages in rural areas leading to inability to charge phones were also mentioned as potential barriers to service delivery with Peek. Provision of HCPs with a battery-powered charging system and backing up data were given as potential solutions.

You know the challenges of network in Kenya, the downs, you know sometimes it just disappears in some areas and especially in the villages, in the remote areas.

Stakeholder #2

## Discussion

This qualitative study offers a comprehensive understanding of the potential value and barriers to the deployment of the smartphone-based eye examination system, Peek, in developing countries with limited coverage of ophthalmic services. Peek as a stand-alone system is useful; however, in conjunction with smartphone functionalities it offers a highly desirable advantage. To date, studies evaluating mHealth have mainly assessed basic use of MP technology with limited evidence on the value of using smartphones for health care [7,12]. This study showed that Peek is an acceptable examination kit for HCPs, patients, and stakeholders and has the potential to strengthen the delivery of eye care in resource-poor contexts. The study has also illustrated the potential challenges and facilitators that are likely to affect the adoption and deployment of Peek.

The analysis of the user, task, technology, and environment gives an overall understanding of the context in which Peek is being evaluated. The patient diversity, patient demographics, and the HCP roles and experience utilized in this study are considered to be the representative environment for which Peek has been designed and in which it will likely be deployed. Most HCPs had limited ophthalmic specialist training, serving as CHVs with experience restricted to the year in which the Peek study was undertaken. This has provided useful insights, because if Peek were to be deployed, it is likely that CHVs will be recruited because of the shortage of ophthalmic professionals in developing countries.

Overall, HCPs demonstrated a good understanding of the utility of Peek, that is, its task. The analysis of attitudes toward technology revealed that HCPs, patients, and stakeholders perceived the population as being familiar with MPs and receptive to them being used. These views reflect the increasing penetration of MPs and more specifically smartphones in Kenya. This enthusiasm for MPs has been greatly influenced by a number of initiatives: M-Pesa's money transfer initiative that has driven MP usage in the remotest of settings and Safaricom's initiative to make smartphones more affordable through the introduction of cheaper android devices, which have led to increasing smartphone subscriptions [11].

The analysis of the context also revealed significant barriers to seeking and accessing ophthalmic services in the current HS. Both HCPs and patients felt that there was a rural–urban disparity with almost no established services in rural settings. This was reported to lead to patients having to travel long distances, having to encounter long waiting times at overburdened government facilities, and having a lack of awareness about timely detection and treatment.

Peek was found to be acceptable to patients, all of whom expressed being satisfied with Peek. Moreover, the analysis revealed that contentment with the service was often related to the quality of service provision. Most participants supported the use of Peek because it was perceived to be fast and convenient and to be able to reach a larger population in need, in addition to overcoming the aforementioned barriers. Peek is also deemed to have generated a lot of interest among the communities and is therefore an opportunity for increasing awareness of eye health within the population. Although limited, a handful of studies have shown the value of mHealth initiatives in creating awareness, for instance, in general health, HIV/AIDS, and women's health in low-income countries [34].

The analysis of the usability of Peek based on predefined usability dimensions demonstrated that per HCPs' perceptions, Peek generally fulfills the criteria for all dimensions assessed. These included efficiency, effectiveness, learnability, and operability and flexibility. In addition to the usability of Peek, the analysis confirms that Peek is acceptable to HCPs. This was demonstrated by their perceived ability to use Peek easily, fulfill their role, and meet the challenges of ophthalmic provision.

An analysis of the views of HCPs and stakeholders using a model adapted from relevant literature showed the value of Peek in strengthening the HS's ability to provide eye services [30-33]. Peek was perceived to be a capabilities enhancer for HCPs through the provision of diagnostic and decision support. This has already been introduced as an important feature of mHealth initiatives in supporting HSs as proposed in current literature [9,33]. The possibility of using Peek as a screening tool is also discussed under this theme, and its success is thought to be dependent on being able to prove its accuracy, sensitivity, accessibility, and ability to offer a high standard of service delivery. It is therefore vital that these qualities are satisfied in addition to other criteria required for enrolling a screening service before Peek can be rolled out for this purpose [38]. A qualitative study of the accuracy of the tool has been carried out alongside this qualitative study, which has proven its accuracy, repeatability, and consistency as a vision-testing tool. Another study is also underway to determine its suitability as a screening tool in children at school.

Peek's value in creating opportunities that help in supporting health care delivery was also highlighted. These included offering eye care closer to patients and enabling monitoring and surveillance. Additionally, Peek was deemed to be a social enabler and improved communication between providers themselves as well as with their patients. Another theme highlighted was knowledge creation and development of skills by offering training opportunities. The outlined benefits of Peek show its potential value in supporting CHVs in providing a high standard of care through its inbuilt functions, because these support decision making as well as communicating with qualified ophthalmic professionals who can offer advice remotely. Other studies of mHealth in developing countries have demonstrated the value of MPs in tackling the current barriers to service provision and improving the range and quality of services offered by CHVs [34,39,40]. Moreover, these benefits are likely to play an important role in the near future, with the increasing double burden of disease in Africa where chronic ophthalmic conditions, such as glaucoma, age-related macular degeneration, and diabetic retinopathy, are also likely to become more prevalent. As a consequence, the need for Peek to be offered within a well-coordinated HS that is capable of screening for and managing these conditions as part of secondary prevention efforts is likely to become increasingly essential.

Given the paucity of studies and established guidelines on large-scale implementation of mHealth, the views of all parties gathered during this qualitative analysis assisted in understanding the challenges and facilitators in deploying Peek.

This analysis brought out several common themes that highlight key considerations that are likely to affect adoption of Peek. Many of the challenges reported are similar to those mentioned in previous mHealth studies [41]; however, several unique considerations were also revealed that are specific to Peek and the context in which it is likely to be implemented. The themes that emerged included the need for (1) government support and involvement in deployment, (2) building capacity to train HCPs and maintenance of Peek, (3) maintaining a high standard of care and good communication about the purpose of using Peek with all patients, (4) community mobilization, (5) increasing capacity to manage increasing demand for eye treatments, (6) ensuring general eye health awareness and linking with primary health care, (7) ensuring data protection, (8) ensuring accessibility to smartphone technology at low cost, and (9) infrastructural support such as mobile charging systems and improved network coverage. These considerations serve as guidance for the future implementation of Peek.

Although previous mHealth studies have been conducted on a small scale, a review of literature has shown that there is a clear opportunity for successful mHealth interventions when proven to be acceptable, accessible, easy to use, affordable, appropriate to the local context, and integrated within the HS [8,39,42-46]. Peek therefore shows promise for success.

### Limitations

Since the qualitative analysis was carried out on data that had been collected retrospectively, there was limited opportunity for an iterative process whereby initial data analysis can guide further interviews. Moreover, in an attempt to answer specific predefined objectives, data were collected from semistructured interviews, which limited open-ended questions. Additional open-ended questions would have allowed for a more in-depth exploration of themes.

### Conclusion

The analysis of context illustrated the perceived importance of addressing rural-urban disparity and thereby the need to increase access and coverage of ophthalmic provision. The key barriers highlighted were cost, distance, time, and lack of awareness of the importance of timely detection and treatment. From the analysis of patient, stakeholder, and user views regarding Peek, it can be concluded that Peek offers an acceptable solution to overcoming barriers to access to eye care, fulfills the criteria for usability for HCPs, and acts as a means to strengthen eye care delivery. Peek is perceived to be valuable predominantly in increasing coverage in rural settings, thereby contributing to the third global goal for sustainable development [47]. As proposed by the HCPs and stakeholders, it is also likely to have a bearing on reducing burden on ophthalmologists who with the help of CHVs using Peek can now work remotely through task shifting. To successfully deploy Peek and achieve universal coverage, it is considered imperative to build a sustainable model by integrating and working with the government, local communities, and NGOs. Ongoing research would be required to evaluate the processes of deployment and to assess whether the benefits outlined translate to improved eye health outcomes and public health indicators.

## Abbreviations

CHV: community health volunteer
HCP: health care provider
HS: health system
mHealth: mobile health
MP: mobile phone
NGO: nongovernmental organization
Peek: Portable Eye Examination Kit
SMS: short message service
TAM: Technology Acceptance Model
VA: visual acuity
WHO: World Health Organization

## Appendix

Multimedia Appendix 1 Peek application screenshots.
