# Supplementary material for: Acceptability, Usability, and Views on Deployment of Peek, a Mobile Phone mHealth Intervention for Eye Care in Kenya: Qualitative Study
Source: JMIR Mhealth Uhealth. 2016 May 9;4(2):e30. doi: 10.2196/mhealth.4746 (PMC4877502; doi:10.2196/mhealth.4746)
Supplement: Multimedia Appendix 1 [file mhealth_v4i2e30_app1.pdf]

Eye exams on your mobile

Visual Acuity
Test distance vision

SightSim™
Simulate visual acuity

Tutorial
Learn how to use Peek

Units

- ○ logMAR (1.0)
- ◉ Snellen - metric (6/60)
- ○ Snellen - imperial (20/200)

Instructions

- [x] Show full instructions on next test

Feedback

- [ ] Send anonymous feedback

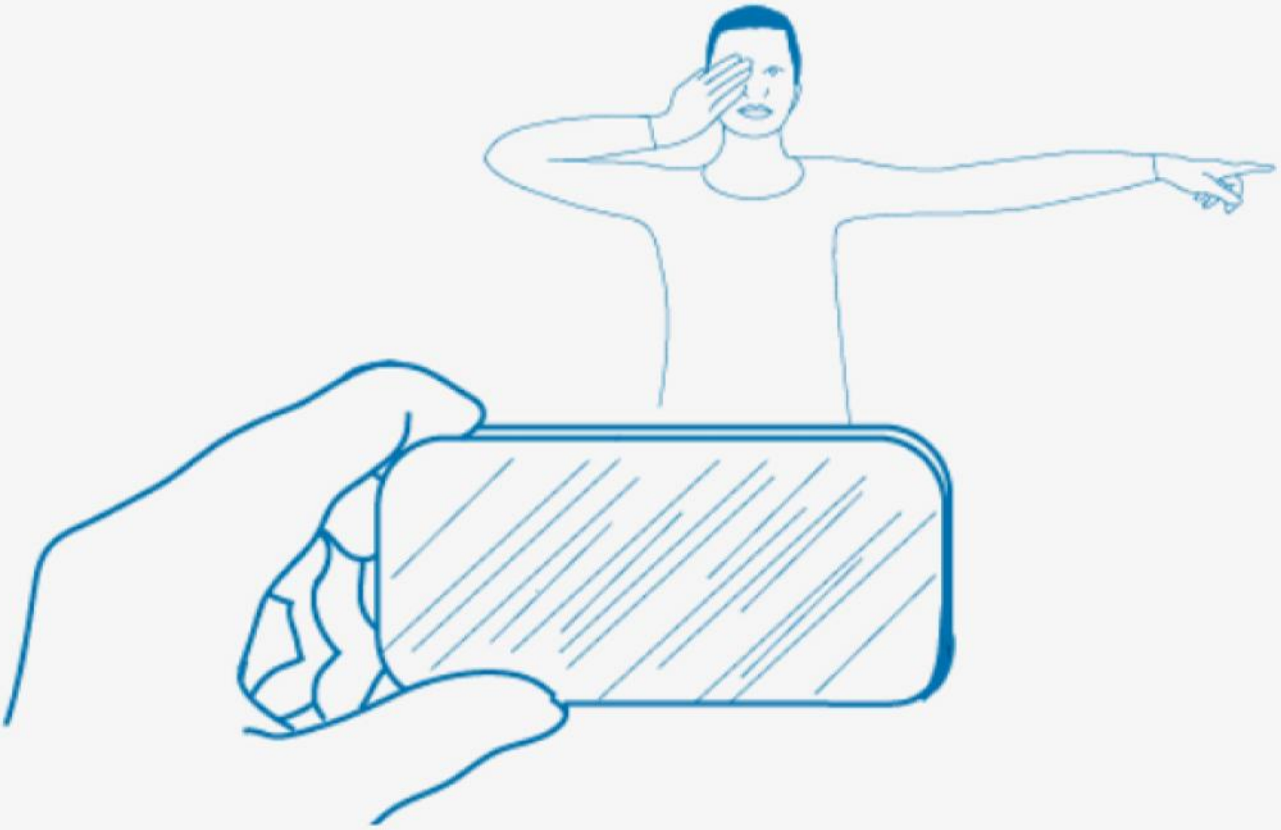

Ask the client to show you which way the legs or the E are pointing.

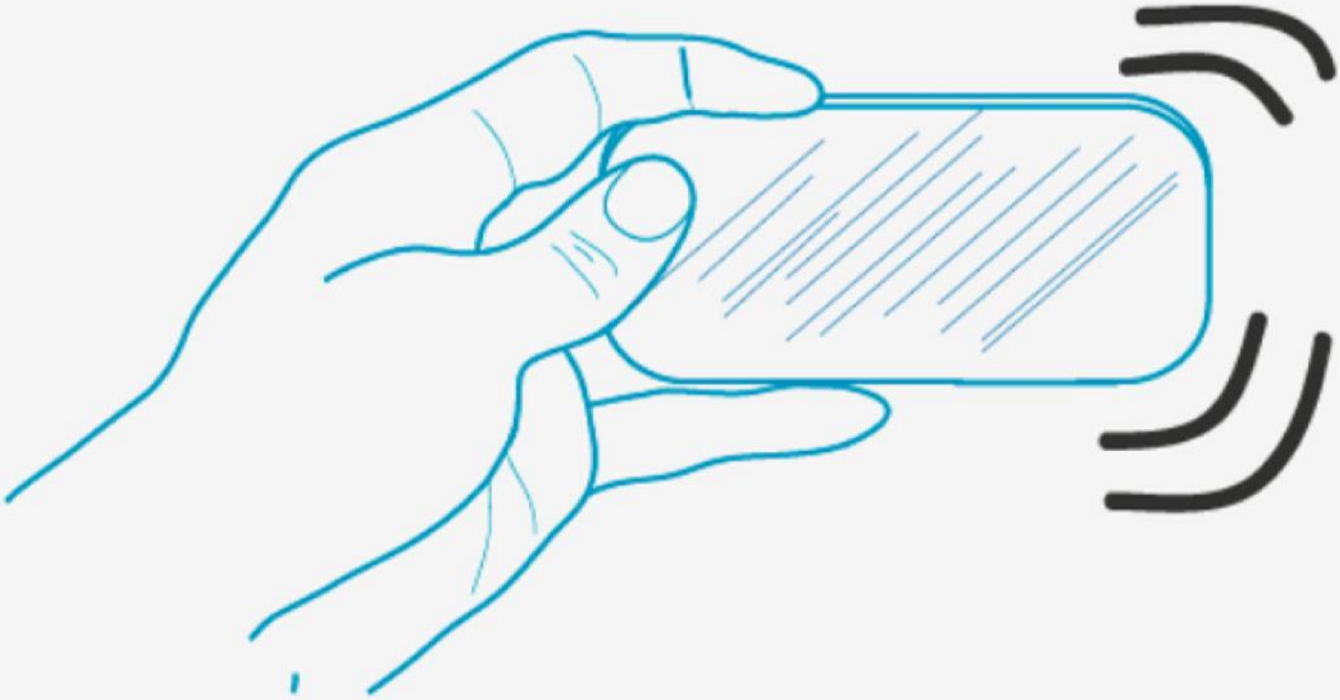

When the test is finished, the phone will vibrate and you will hear an alert. The results will be shown on the screen.

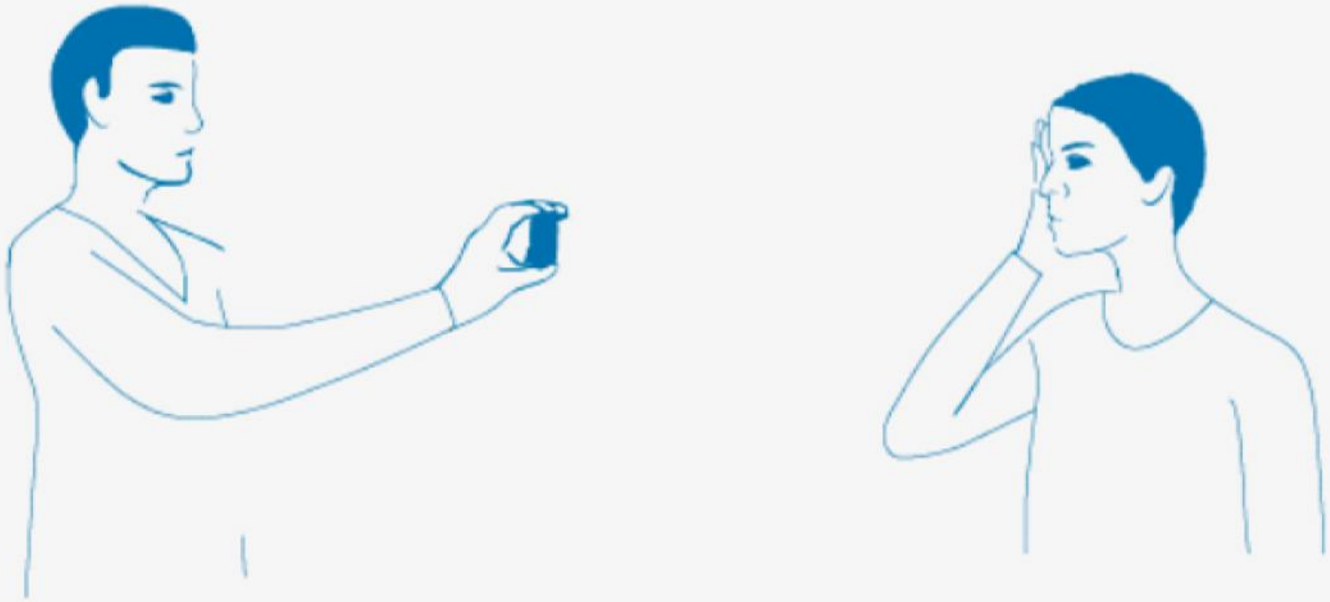

Hold the phone at the clients eye height at a distance of 2 meters.

BACK DONE

Ш

The tested vision level is 6/60. We can show an approximation of how this is affecting your view of the world with SightSim.

SIGHTSIM™ DONE

Normal Visi

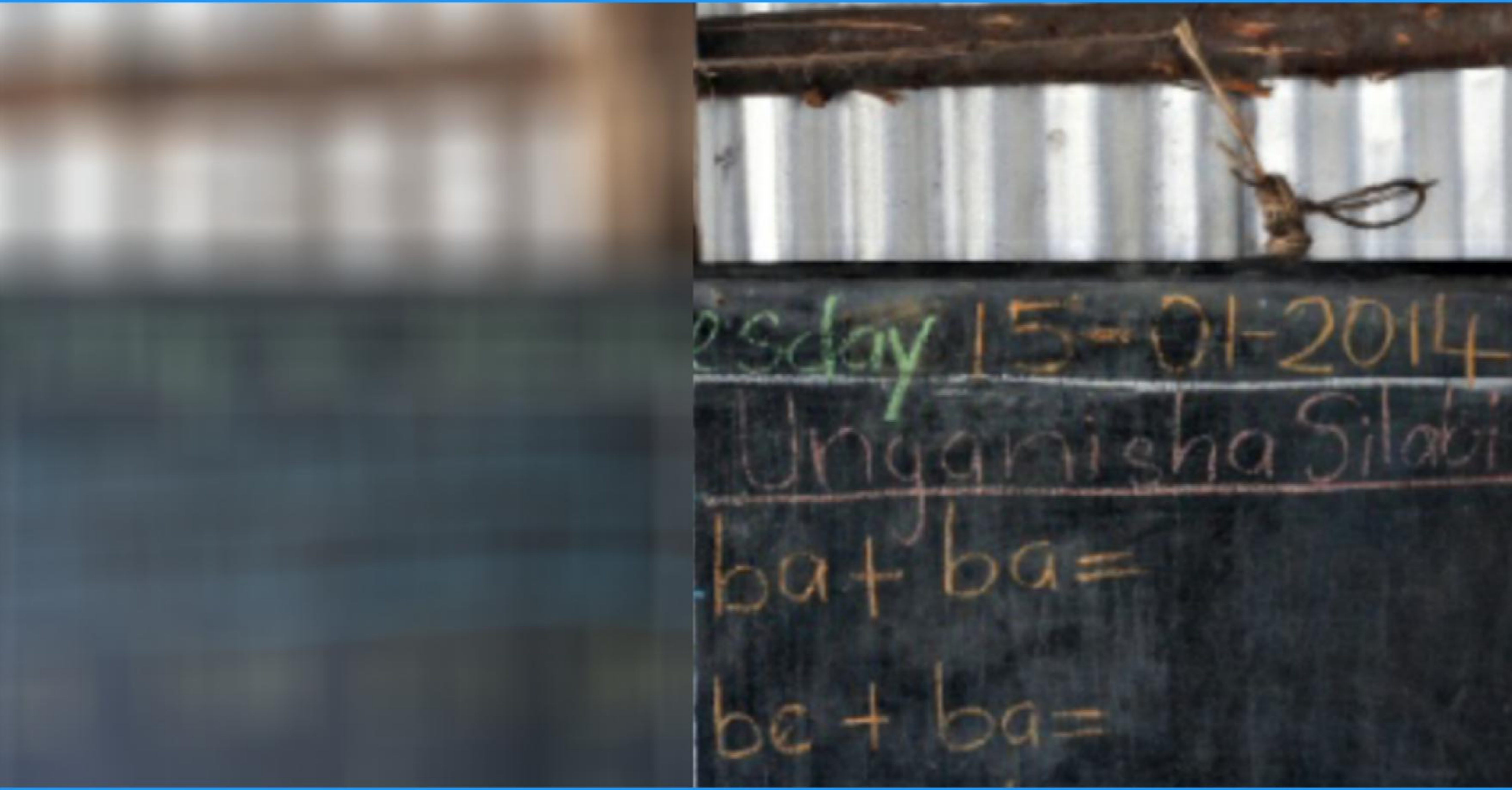

Blackboard
